# Mechanisms of body fat distribution and gluteal-femoral fat protection against metabolic disorders

**DOI:** 10.3389/fnut.2024.1368966

**Published:** 2024-03-25

**Authors:** Maha Alser, Khaled Naja, Mohamed A. Elrayess

**Affiliations:** ^1^Biomedical Research Center, Qatar University, Doha, Qatar; ^2^QU Health, Qatar University, Doha, Qatar

**Keywords:** metabolic protection, prediabetes, type 2 diabetes, cardiovascular diseases, body fat distribution, thigh fat

## Abstract

Obesity is a major health problem that affects millions of individuals, and it is associated with metabolic diseases including insulin resistance (IR), type 2 diabetes (T2D), and cardiovascular diseases (CVDs). However, Body fat distribution (BFD) rather than crude obesity is now considered as a more accurate factor associated with these diseases. The factors affecting BFD vary, from genetic background, epigenetic factors, ethnicity, aging, hormonal changes, to lifestyle and medication consumptions. The main goal of controlling BFD comes from the fact that fat accumulation in different depots has a different effect on the overall health and metabolic health of individuals. It is well established that fat storage in the abdominal visceral depot is associated with metabolic disorder occurrence, while gluteal-femoral subcutaneous fat depot seems to be protective against these diseases. In this paper, we will summarize the factors affecting fat distribution. Then, we will present evidence connecting gluteal-femoral fat depot with protection against metabolic disorders including IR, T2D, and CVDs. Finally, we will list the suggested mechanisms that lead to this protective effect. The abstract is visualized in Graphical Abstract.

## GRAPHICAL ABSTRACT

Graphical abstract showing a summary of the factors affecting body fat distribution, the difference between abdominal and gluteal-femoral obesity on metabolic disorders progression, and the protective mechanisms of gluteal-femoral fat.

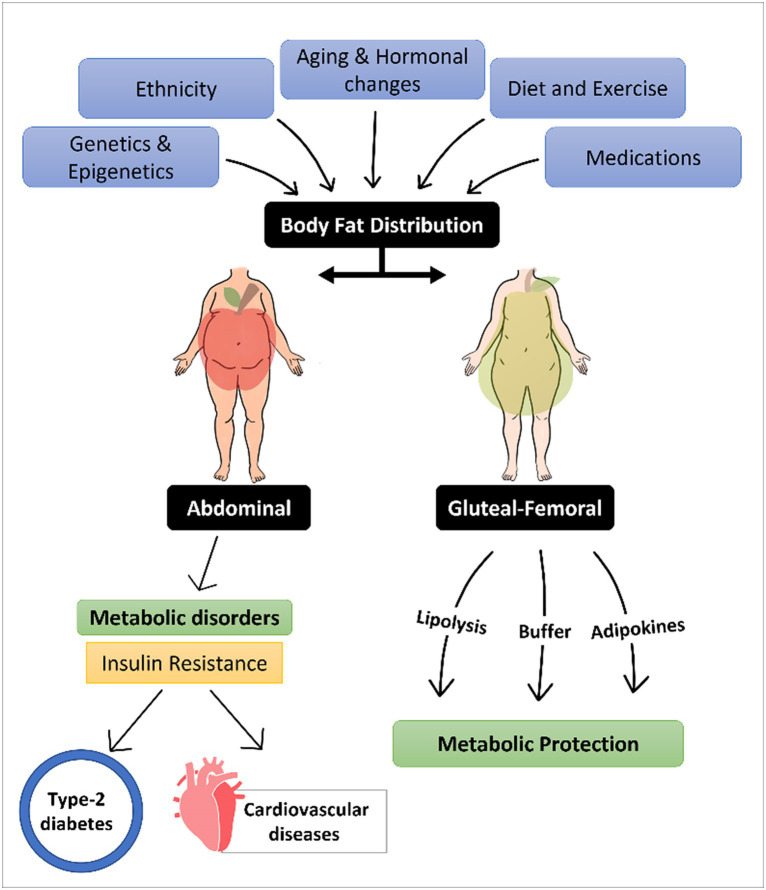

## 1 Introduction

Obesity is a major public health problem, affecting millions of individuals worldwide. According to World Obesity Atlas (1), the proportion of the obese population (BMI ≥ 30) will reach 17% in 2025, and 20% in 2030 affecting more than 1.5 billion people. It is noteworthy to mention that COVID-19 pandemic has worsened the obesity epidemic, and the restrictions from 2020 to 2022 have increased the risk of weight gain by increasing sedentary and unhealthy dietary behaviors, and significantly reduced access to health care (2).

Obesity is a complex multifactorial disease, meaning it is caused by multiple interacting factors, including genetics, environmental, and behavioral factors (3). The long-term serious effect of obesity lies in the increasing likelihood of many diseases, including insulin resistance (IR) and type 2 diabetes (T2D) (4), chronic kidney disease (CKD) (5), non-alcoholic fatty liver disease (NAFLD), and some cancers (6). Additionally, both obesity and T2D increase the prevalence of cardiovascular disorders (CVDs) (7). Although obesity and BMI represent a good indicator of the overall metabolic health profile, studies have shown that the distribution of body fat into fat depots plays a more important role in the co-morbidity of the metabolic diseases (8). This indicates that BFD measurements along BMI need to be considered to assess metabolic health. These measurements include simple waist to hip ratio, where the waist circumference (reflecting abdominal adiposity) is divided by the hip circumference [reflecting gluteal femoral (GF) fat adiposity]. Other more accurate measurements include body imaging to measure actual fat depot sizes.

Adipose tissue functions not only as a fat storage organ, but also as a dynamic organ (9); therefore, fat storage in different depots have diverse impact on human health. In fact, each fat depot contributes differently as metabolic and endocrine organ, leading to different levels of metabolic disorders (10). It is well established that over-accumulating fat in the abdominal fat depot is associated with metabolic disorders; Increases of abdominal adiposity as opposed to overall adiposity, is associated with increases in the likelihood of getting T2D and CVDs 4 (11). Some studies showed hip circumference and thigh fat adiposity alone as an independent indicator of obesity-related metabolic risks (12). More recent evidence suggests that GF fat depot shows a protective role against these metabolic disorders; fat storage in the GF depot is preferred over storage in the abdominal depot to gain a metabolically healthy state (13–16). These opposing relationships reflect the unique intrinsic characteristics of different fat depots.

Explaining the factors that lead to distinct BFD and resolving the mechanisms behind the protective role of some depots, may help in finding novel therapeutic venues for preventing or treating obesity-related diseases. In this review, we summarized the important factors affecting BFD, whether uncontrollable (genetics, epigenetic factors, age, and hormonal profile) or controllable (diet, exercise, and consumption of certain medications). Then, we compiled detailed evidence supporting the protective role of the GF fat depot against metabolic disorders, including IR, T2D, and CVDs. Finally, we summarized the reported possible mechanisms explaining how this protection is accomplished.

## 2 Fat depots and gluteal-femoral fat biology

**Figure 1:**
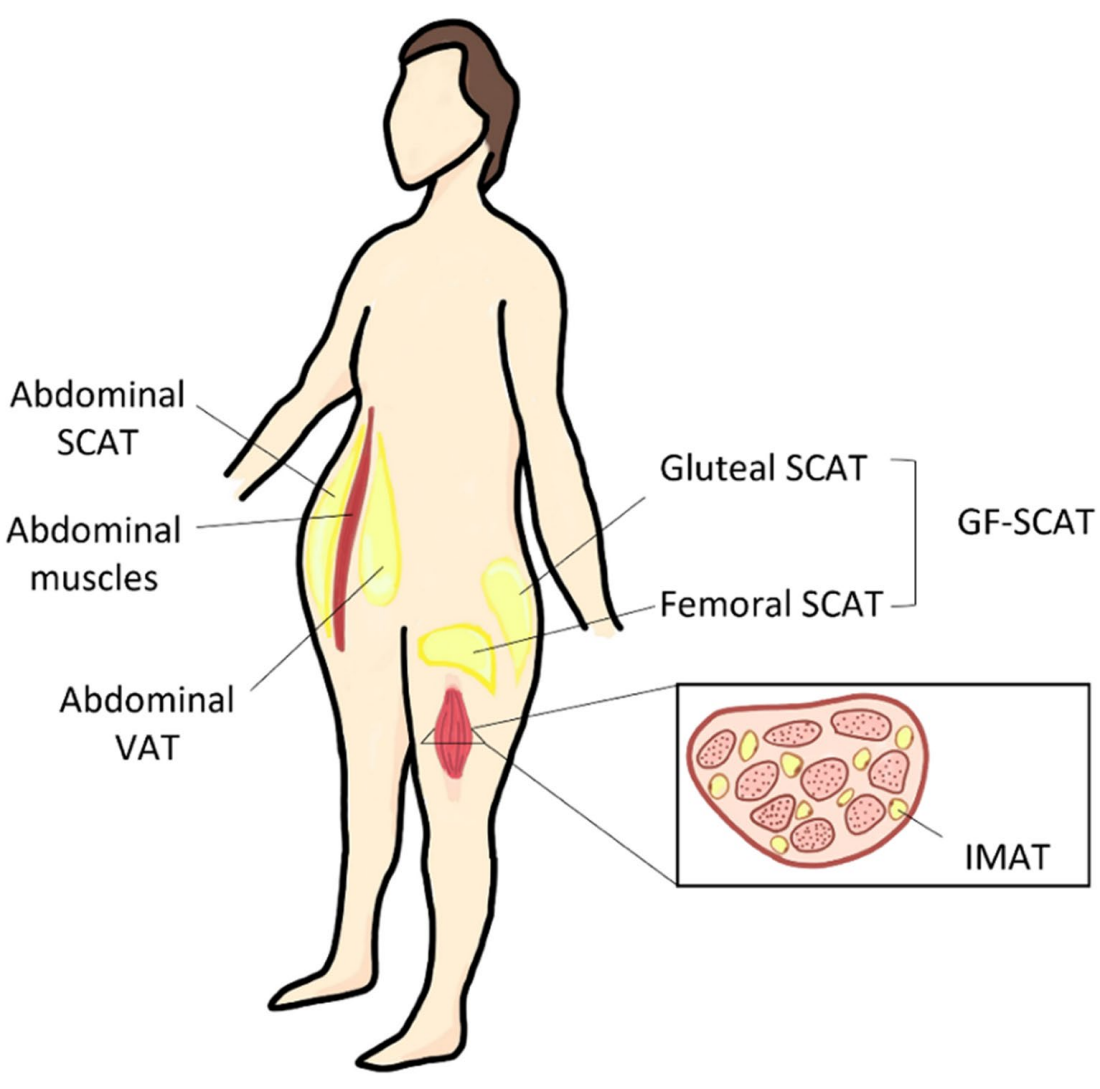
Different body fat depots and their anatomical location. SCAT, subcutaneous adipose tissue; VAT, visceral adipose tissue; GF, gluteal-femoral adipose tissue; and IMAT, intermuscular adipose tissue.

Adipose fat depots are the specific location where the fat tissue is built and located in the body. The human body stores fat in 2 anatomically and physiologically distinct fat depots; subcutaneous adipose tissue (SCAT) and visceral adipose tissue (VAT). SCAT is located between the skin and superficial musculature, and it can be further classified into superficial SCAT and deep SCAT (17). The main depots where SCAT is stored are the inguinal region (GF regions), and the anterior and the back of the abdominal wall as shown in Figure 1. On the other hand, VAT depot, also known as intra-abdominal fat, is the fat tissue stored within/between the visceral organs, such as liver and intestines (8). Physiologically, VAT is more vascular, innervated, and contains less preadipocyte differentiating capacity, greater percentage of large adipocytes and larger number of inflammatory and immune cells as compared to SCAT (18). Individuals with enhanced abdominal obesity, more specifically with high VAT depot content are referred to apple shaped, as compared to pear shaped individuals that express higher GF content (19), as illustrated in Graphical Abstract.

The GF fat as a combined depot is referred to as gluteal-femoral fat (GF fat/GF adipose tissue) as shown in Figure 1. In literature, GF fat is referred to as thigh, hip, or lower body fat, and could be further classified into different depots. These depots differ in their biology, histology, and physiological role. GF subcutaneous adipose tissue (GF-SCAT) is the fat tissue stored under the skin of the lower body part. Another thigh fat depot is the thigh intermuscular adipose tissue (thigh IMAT) illustrated in Figure 1, which is considered as an ectopic fat depot; fat stored in tissues other than specialized adipose tissue, skeletal muscle tissue in this case (8). In the following section, we discussed the factors affecting BFD, favoring specific depots over the others.

## 3 Factors affecting body fat distribution

Body fat distribution (BFD) is affected and mediated by multiple factors. Genetic background plays a significant role in determining BFD. Studies have shown that certain genes are associated with increased abdominal fat, while others are associated with increased fat in the hips and thighs (20–25). Epigenetic differences, which impact obesity, may also have an influence on body fat distribution (26–28). Ethnicity, aging, and hormonal changes also play a role in BFD (7, 29, 30). On the other hand, certain factors can be induced to manipulate BFD. Diet restriction and exercise can lead to fat reduction in the abdominal region (31). Certain medications, such as those used to treat diabetes or high blood pressure, can also lead to a redistribution in body fat (19). The factors that affect BFD are summarized in Table 1.

**Table 1:** Summary of the factors affecting body fat distribution.

Factor	Description	References
Genetic	Many loci associated with BFD, that are greatly distinct from those of BMI and obesity risk. *S70C* variant in Coiled-Coil Domain Containing 92 gene is associated with decreased visceral fat (VAT) and increased leg fat (GF-SCAT). Dynein axonemal heavy chain 10 (*DNAH10*), Plexin-D1 (*PLXND1*), R-spondin 3 (*RSPO3*), and lysophospholipase-like 1 (*LYPLAL1*) genes are high determinants of BFD. HOXA5 expression is upregulated in abdominal subcutaneous adipose tissue compared to gluteal subcutaneous adipose tissue.	(20–22, 24, 26, 29)
Epigenetic	DNA promoter methylation of IRS1 in human adipose tissue is related to fat distribution.	(32)
Ethnicity	Asian ethnic group has more visceral fat as compared to Europeans. Asian women have less GF-SCAT, and greater abdominal VAT compared to Caucasians and African Americans. Mexican Americans have more visceral obesity as compared to non-Hispanic white Americans.	(13, 29, 33)
Aging and hormonal changes	Aging leads to changes in BFD, with a shift from subcutaneous to visceral fat, especially in men. Estrogen accumulates fat in GF-SCAT depot, and not in abdominal fat depot. Menopausal transition is associated with fat accumulation in the central body depot (abdominal) as compared to thigh depot.	(7, 30, 34)
Diet and exercise	Aerobic exercises and dietary restrictions have been found to be independent factors contributing to a substantial decrease in visceral adipose tissue (VAT). Combined effect of exercise and diet may have more effect on BFD. The Mediterranean diet leads to mobilizing ectopic fat to other healthy depots, while exercise only leads to ectopic fat loss.	(31, 35–37)
Medication	Glucocorticoid VAT mass through lipolysis stimulation by activating the hormone-sensitive lipase and increasing catecholamine sensitivity. Metformin significantly decreases abdominal obesity, including SAT and VAT reduction. Thiazolidinediones were associated with a reduction of VAT/SAT ratio in a dose-dependent manner. SGLT2 inhibitors were associated with a significant reduction of liver and pancreatic fat. Glucagon-like Peptide Receptor Agonists (GLP-1) lead to a significant reduction in VAT. New GLP-1/GIP/glucagon triple agonist therapy normalizes body weight. DPP-4 inhibitors, in combination with metformin are associated more reduction in visceral fat compared to metformin alone.	(38–45)

## 4 Gluteal-femoral fat protection against insulin resistance

Insulin resistance (IR) is a defined as a state where the body cells become irresponsive to the physiological insulin levels secreted in the blood (46). IR is a risk factor for many chronic metabolic disorders. The major consequence of IR is the progression of type 2 diabetes, where individuals diagnosed with IR are expected to develop T2D in 5 ~ 10 years (46). T2D is highly diverse disease. To personalize the treatment of T2D, a study attempted to classify the disease into clusters, depending on six variables: including glutamate decarboxylase antibodies, age, BMI, HbA1c, and homoeostatic model assessment 2 estimates of β-cell function and insulin resistance. The significance of this classification is to generate a way to estimate the risk of diabetic complications, which they successfully showed in their study. According to the researchers, one cluster (highest insulin resistance) showed more association to kidney disease, another cluster (insulin deficient) showed the highest risk of retinopathy, while a third cluster associated with higher fat liver, indicating a relevance of VAT to IR (47).

Previously, obesity was believed to be a major leading cause of IR. However, recent investigation showed that abdominal obesity is positively associated with IR, while considerable debate remains concerning the potential of a positive effect of thigh fat in metabolic protection against IR progression (19). This theory explains the metabolically healthy obese individuals and how their bodies escape the negative metabolic consequences of obesity (48). Thigh fat is protective against IR; a study demonstrated that lower thigh SCAT and higher VAT both contribute to insulin resistance profile. The study was conducted on a cohort from the general population, it demonstrated that high thigh SCAT alone or low VAT alone were positively associated with IS state while Individuals with combination of high SCAT and low IMAT had the highest IS state among all groups (49). A more recent study investigated both thigh fat depots (thigh SCAT and thigh IMAT) in a female cohort. They showed that higher thigh-SCAT and lower thigh-IMAT are associated with insulin sensitivity (14). The same findings were supported with studies done on children obese cohort (age of 7–17 years old), where a study suggested a protective effect of thigh fat, and its association to a healthy metabolic profile [more favorable HOMA-IR score, triglyceride serum level, systolic blood pressure (SBP), C-reactive protein (CRP), resistin, high-density lipoprotein (HDL), cholesterol, adiponectin, and blood lipid profile] (15). Whereas another study conducted on postmenopausal women suggested that thigh fat was associated with a favorable metabolic profile, including serum triglycerides, HDL-cholesterol, and various IR markers (50). Due to the reported association between thigh fat and protection against IR, a study suggested that regional adiposity, including thigh fat size could be used as a predictive way to predict the risk of developing IR. They reported that each increase in thigh fat led to a 59% decrease in the odds of becoming insulin resistant, independently from confounders (age, BMI, visceral adiposity, and gender) (51).

It has been reported that a larger gluteal femoral fat is associates with lower prevalence of IR, prediabetes, and dyslipidemia (52). However, as explained in the previous section, not all thigh fat seems to be protective. A study assessed critical biomarkers and how their levels are different among individuals with different BFDs. The findings suggested a significantly lower insulin resistance and triglycerides levels in individuals with higher GF fat (13), but according to Snijder et al. (52), only thigh-SCAT was found to be associated with favorable metabolic profiles. Higher thigh-SCAT was shown to be associated with lower transformed triglycerides (in both genders), higher HDL-cholesterol, and lower blood glucose level (in males only).

The revised studies showed how GF-SCAT is beneficial as a protective fat depot against metabolic disease. In contrast, IMAT is an ectopic depot for fat storage. In contrary with SCAT, fat deposition in IMAT is highly associated with increase IR and T2D (53). One suggested mechanism of how IMAT promotes IR progression is because fat accumulation within the thigh muscular tissue leads to reduced blood circulation, and decreased insulin uptake efficiency by the muscle cells (54). These studies confirm the association between GF SCAT levels, not GF IMAT, and developing IR leading to multiple metabolic disorders, including type 2 diabetes and cardiovascular disease as explained in the following section.

## 5 Gluteal-femoral fat cardio protection

Cardiovascular diseases (CVDs) are a general term that describes a range of pathologies affecting the health of the heart, the blood vessels, or the pericardium. It is critical to study the risk factors affecting and leading to CVDs as these diseases can be fatal. Previous studies have shown a clear association between BFD and the risk of developing CVDs (19, 50, 55). While increased fat storage in the abdominal depot; mainly ectopic visceral fat is associated with high CVDs progression risk, high GF fat storage was shown to be cardioprotective (56).

The cardioprotective role of GF fat has been confirmed in many studies involving diverse human subjects with different ages, BMIs, and diseases. As early as 1991, thigh fat has a negative association with cardiovascular diseases was first hypothesized. High thigh fat was shown to contribute to lipoprotein plasma profile; an indicator of lower risk of cardiovascular diseases (57). Following that finding, more research groups started showing the same correlation among different models. Accumulating evidence shows an association between increased lower body adiposity and GF fat and healthy lipid profile. Many studies showed evidence that elevated GF fat is associated with better serum lipid levels, including lower low-density lipoprotein-cholesterol profile, and higher high-density-cholesterol levels, which are indicators of improved cardiovascular health^8^. Van Pelt et al. (50) showed an enhanced triglycerides level in individuals with higher GT fat as compared to others with the same BMI.

In addition to improved serum lipid profile, increased GF mass has been shown to be directly associated with enhanced vascular health, including lower aortic calcification, decreased progression of present aortic calcification, as well as decreased arterial stiffness in a women cohort (58). Furthermore, studies showed a direct effect between GF fat and cardiac diseases. Yusuf et al. (59), conducted a study that involved 27,000 participants, they showed a negative association between hip circumference and the risk of myocardial infarction. Another study confirmed that a larger hip circumference is associated with lower risk of developing coronary heart disease in a population involving both genders (60). Shay et al. (16) reported that women characterized with higher GF fat content and suffer from type-1-diabetes have shown less risk of developing coronary artery disease, which was not shown in men.

The protective effect of GF fat against CVD risk persists even with aging. A study was conducted on healthy elderly women, they confirmed a strong association between GF-fat and protection against vascular damage (55). These studies confirm the tight association between GF levels and the risk of CVDs. More studies were well revised by Manolopoulos et al. (61). In the following section, we will revise the reported mechanisms that explain this association, as well as the possible mechanisms that need more investigation to be proven.

## 6 Mechanisms of gluteal-femoral fat metabolic protection

As mentioned previously, thigh fat shows protective properties against IR and CVDs. While the mechanism behind this association remains not fully understood, research suggests three major mechanisms by which this protection is achieved. These mechanisms ultimately enhance metabolic health and lower the risk of developing IR and CVDs.

### 6.1 Catecholamine-mediated lipolysis

Lipids are mobilized by adipocyte lipolysis, a fundamental process of hydrolyzing triacylglycerol to fatty acids for internal or systemic energy use. The rate of lipolysis is low in the subcutaneous femoral-gluteal region, intermediate in the subcutaneous abdominal region and high in the visceral region (62). Indeed, abdominal adipocytes showed β-adrenergic lipolytic sensitivity 10–20-fold greater than gluteal adipocytes; this is due to an increase in the total number of β-adrenoceptors in this depot (63).

A study showed that the steady-state mRNA levels of beta-adrenergic receptors BAR 1 and BAR 2 were about twice as high in abdominal as in gluteal adipocytes of men and women (*p* < 0.01) explained by an increased expression of the genes that encode for *BAR 1* and *BAR 2* (64). In women, variation in the affinity properties of the α-2 adrenoceptor is an additional factor. Abdominal adipocytes showed a 40 times lower α-2 adrenergic antilipolytic sensitivity than did gluteal adipocytes (63).

The decreased action of β-adrenergic receptors and increased activity of α_2_-adrenergic adrenoceptors in combination with defects in hormone sensitive lipase function inhibits the lipolytic effect of catecholamines in subcutaneous fat cells, whereas increased activity of β-adrenergic receptors and decreased activity of α_2_-adrenoceptors increases the lipolytic response in visceral fat cells. These abnormalities in catecholamine function promote release of free fatty acids from the visceral fat cells to the liver through the portal system and might cause several of the metabolic complications to upper-body obesity (62).

### 6.2 Gluteal-femoral adipose depot acts as a buffer to control excess lipids

The protective properties of GF fat against insulin resistance and cardiovascular diseases relies mainly on the fact that it is physiologically different from other fat depots, especially abdominal fat depot. This mechanism suggests that the protection is due to the nature of thigh depot. Most of the thigh stored fat is stored in the subcutaneous fat depot (GF SCAT) rather than the ectopic thigh depot (GF IMAT). Fat storage in the GF SCAT depot keeps the excess fat away from the visceral region, which is linked to high risk of metabolic disorder progression. Additionally, accumulating evidence from *in vivo* and *in vitro* studies showed a differential regulation of GF SCAT fat mechanisms of fatty acid uptake and release at the adipocyte level. In case of increased dietary lipid intake, GF SCAT depot becomes a long-term store of free fatty acids, preventing them from accumulating in ectopic fat depots, especially in the viscera and within vital organs (65). This is likely since GF fat depot has a relatively high lipoprotein lipase activity as compared to other depots, making it a better reservoir of excess circulating fatty acids. This storage prevents lipid overflow within abdominal visceral depot, causing reduced lipotoxicity, as well as decreased risk of IR and CVDs on the long run (13).

This theory simply shows that the protective association is due to the absence of the risk, rather than an actual protection mechanism (66). However, more recent research has shown other mechanisms that contribute to this protection. According to Tran et al. (67), the protective effect of GF SCAT is not due to its depot, but rather to intrinsic differences between this depot and visceral fat depot. This research group assessed how transplanting GF SCAT from healthy mice to the visceral depot of metabolically unhealthy mice, which lead to enhanced metabolic profile in the recipient mice, including decreased total body weight, fat mass, blood glucose, and insulin levels. These findings recommend that the difference is not as simple as GF SCAT working as a buffer to control excess fat from getting stored in the visceral region. GF SCAT certainly plays a more crucial role at the physiological level to gain this protection and improve insulin sensitivity. Recent studies suggest that GF SCAT is intrinsically different from other depots as is it plays a critical role as an endocrine organ that is associated with a protective adipokine profile. The protective role of GF SCAT as an endocrine organ is discussed in detail in the next section.

### 6.3 Gluteal-femoral adipose depot contributes to lipoprotein profile and secretes protective adipokines

Lipoproteins are molecular structures that consist of proteins associated with fat with the main function to transport lipids (triglycerides and cholesterols) throughout the body (68) and serve an important role in metabolic health. The two major types of lipoproteins are the high-density lipoprotein (HDL) and low-density lipoprotein (LDL), which are often referred to as “good” and “bad” cholesterol in the metabolic disorder context. Terry et al. (57) showed a positive correlation between thigh fat and HDL levels, and where the first to suggest that thigh fat contributes to plasma lipoprotein profiles and might predict lower risk of metabolic disorders, mainly CVDs.

Conversely, Adipokines are special cytokines secreted by adipose tissue components, including adipocytes and other cells as macrophages. Adipokines (adiponectin and Leptin) are mainly produced by the adipocyte component of the adipose tissue, while interleukins (such as IL-6) are produced by residential macrophages (61). It is known that a healthy adipokine profile is associated with reduced risk developing CVDs. Recent studies suggested that GF depot has a different secretome profile than other depots. It was shown to be associated with elevated levels of beneficial adipokines, and less pro-inflammatory adipokines as opposed to abdominal fat depot.

#### 6.3.1 Leptin

Leptin is a critical adipokine in terms of energy metabolism by controlling energy intake and storage as a fat mass (69). Leptin is predominantly expressed by isolated subcutaneous adipocytes as opposed to omental adipocytes, particularly in women (70). Leptin secretion levels are higher in SCAT as compared to VAT. In terms of mRNA expression of Leptin, no previous studies have compared leptin expression level among different SCATs. However, some studies indirectly suggested that the basal expression level of leptin does not differ among SCAT, and it is the same among abdominal SCAT and thigh SCAT (71). As a secreted adipokine, leptin levels negatively correlate with waist to hip ratio (72). A study conducted by Picó et al. (73) reported that the hormone leptin plays a key role in regulating the size and function of fat depots. Based on that, the authors suggest that targeting the leptin pathway may be a potential therapeutic strategy for managing metabolic disorders (73). All previous studies recommend a differential level of leptin and its association with fat distribution. However, the direct association and definition of leptin level as a mechanism of thigh fat protection is an area that needs more investigation.

#### 6.3.2 Adiponectin

Adiponectin is a hormone, an adipokine associated with insulin sensitivity and inflammation. Low levels of adiponectin are associated with increased risk of several metabolic diseases, including CVDs. Recently, adiponectin levels were shown to be significantly lower in individuals with high abdominal fat depot, while adiponectin levels were higher with high GF fat depot (74). This opposing effect of different depots on differential serum adiponectin levels lead a more recent study to assess that as a suggested mechanism of GF fat cardioprotection (75). Gradidge et al. (75) conducted a cross sectional study on a female cohort. They first confirmed the positive association of GF fat with adiponectin serum levels. Then, they proved a negative correlation between GF fat with triglyceride level and insulin resistance, a critical risk factors of developing CVDs (75). Although the study was not conclusive, it gives insight of a tight association between GF fat, adiponectin serum levels, and CVD risk factors (75). Another study showed that higher thigh fat results in higher adiponectin level, leading to enhanced insulin sensitivity (76). In conclusion, a higher GF fat is associated with higher adiponectin secretion and enhanced metabolic health.

#### 6.3.3 IL-6

Interleukin-6 (IL-6) is an inflammatory cytokine produced by multiple tissues including adipose tissue, mainly by macrophages residing in the tissue (77). The secreted level of IL-6 correlates with obesity, the higher the BMI the higher the IL-6 levels in the plasma (78). In terms of different depots, IL-6 increases with visceral fat mass increase, and is negatively correlated with thigh fat mass (79). A study compared the IL-6 profile among obese individuals expressing different BFD profiles with and without metabolic syndrome. They first showed that BFD explains the difference between the obese participants in metabolic health, which they linked to higher levels of IL-6 blood levels, concluding that individuals with a more favorable BFD (lower VAT and higher thigh IMAT) express a more favorable inflammatory profile and metabolic health as compared to their unhealthy counterparts (same BMI, gender, and age group) (48). There are not enough studies to show a causality link between IL-6 profile and thigh fat protective role, and more investigation is needed in this area.

#### 6.3.4 DPP4

DPP4 plays a major role in glucose metabolism. It is responsible for the degradation of incretins such as GLP-1. A comprehensive proteomic profiling of the human adipocyte secretome identified dipeptidyl peptidase 4 (DPP4) as a novel adipokine that may impair insulin sensitivity in an autocrine and paracrine fashion, the protein levels of DPP4 are fivefold higher in VAT compared with SAT in a cohort of obese patients (80).

## 7 Conclusion

The BFD is a highly accurate indicator of metabolic health due to its association with IR, T2D, and CVDs. Th factors that contribute to fat distribution include genetic background, age, hormonal changes, lifestyle (diet and exercise), and medication consumption. It is necessary to understand BFD, as fat storage in certain depots shows a protective effect against metabolic disorders. The gluteal femoral fat depot is one of these depots. Many reports showed its protection against IR, and its metabolic complications (T2D and CVDs). The mechanisms of this protection include three major mechanisms: first, GF depot demonstrates a lower rate of lipolysis. Second, GF depot acts as a metabolic reservoir of excess lipids, preventing them from being stored in the visceral fat depot. And finally, GF depot has a unique physiological nature of GF in terms of lipoproteins, adipokines, and cytokines secretion, it produces protective cytokines (leptin and adiponectin) in higher levels as compared to other depots, while secreting lower levels of proinflammatory adipokines and cytokines (DPP4 ND IL-6). The protective effect of GF fat is interesting, and the mechanisms behind it is still an active area of research that merits further studying to be fully understood.

## Author contributions

MA: Investigation, Visualization, Writing – original draft. KN: Supervision, Writing – review & editing. ME: Conceptualization, Funding acquisition, Project administration, Supervision, Writing – review & editing.
